# Phone Calls to Retain Research Participants and Determinants of Reachability in an African Setting: Observational Study

**DOI:** 10.2196/19138

**Published:** 2020-09-30

**Authors:** Melvin Draaijer, Samanta Tresha Lalla-Edward, Willem Daniel Francois Venter, Alinda Vos

**Affiliations:** 1 Department of Global Health VU Medical Center Amsterdam University Medical Centers Amsterdam Netherlands; 2 Ezintsha (subdivision of Wits Reproductive Health and HIV Institute) University of Witwatersrand Johannesburg South Africa; 3 Julius Global Health Julius Center for Health Sciences and Primary Care University Medical Center Utrecht Utrecht Netherlands

**Keywords:** retention, loss to follow-up, phone, mobile phones, HIV, ART, South Africa

## Abstract

**Background:**

Long-term retention of research participants in studies is challenging. In research in sub-Saharan Africa, phone calls are the most frequently used method to distantly engage with participants.

**Objective:**

We aimed to get insight into the effectiveness of phone calls to retain contact with participants and evaluated determinants of reachability.

**Methods:**

A cross-sectional study was performed using the databases of two randomized controlled trials investigating different kinds of antiretroviral therapy in HIV-positive patients. One trial finished in 2018 (study 1), and the other finished in 2015 (study 2). A random sample size of 200 participants per study was obtained. There were up to 3 phone numbers available per participant collected during the studies. Participants received a maximum of 3 phone calls on every available number on different days and at different times. Voicemails were left, and emails sent wherever possible. We documented how many calls were answered, who answered, as well as after how many attempts participants were reached. To further increase our understanding of reachability, we conducted a short questionnaire assessing factors contributing to reachability. The study was approved by the Research Ethics Committee of the University of Witwatersrand, Johannesburg, South Africa (reference number M1811107).

**Results:**

In our sample size of n=200 per study, study 1, with a median time of 11 months since the last visit at the research site, had a response rate of 70.5% (141/200) participants while study 2, with a median duration of 55 months since the last visit, had a response rate of 50.0% (100/200; *P*<.001). In study 1, 61.5% (123/200) of calls were answered directly by the participant while this was 36.0% (72/200) in study 2 (*P*=.003). The likelihood of reaching a participant decreased with time (odds ratio [OR] 0.73, 95% CI 0.63 to 0.84) for every year since the last face-to-face visit. Having more phone numbers per participant increased reachability (OR 2.32, 95% CI 1.24 to 4.36 for 2 phone numbers and OR 3.03, 95% CI 1.48 to 6.22 for 3 phone numbers compared with 1 number). A total of 141 of 241 reached participants responded to the questionnaire. Of the 93 participants who had changed phone numbers, 5% (50/93) had changed numbers because their phone was stolen. The most preferred method of being contacted was direct calling (128/141) with participants naming this method followed by WhatsApp (69/141).

**Conclusions:**

Time since last visit and the number of phone numbers listed were the only determinants of reachability. Longer follow-up time is accompanied with a decrease in reachability by phone while more listed phone numbers increases the likelihood that someone can be reached.

**Trial Registration:**

ClinicalTrials.gov NCT02671383; https://clinicaltrials.gov/ct2/show/NCT02671383 and ClinicalTrials.gov NCT02670772; https://clinicaltrials.gov/ct2/show/NCT02670772

## Introduction

Mobile phone use has global penetration, making it accepted as an effective method to reach patients and participants during follow-up in research over the past years [1-3]. In South Africa, 97% of households have access to a mobile phone [4]. However, experience has shown that reachability and accessibility by phone is challenging due to changes of phone numbers. Theft and loss of phones are common, with approximately 40% of participants in a study in Durban, South Africa, reporting that they lost a previously owned phone [5]. These factors result in a loss of connection with the participant and loss to follow-up.

Studies in low- and middle-income settings suffer from low rates of retention. One study in Togo including 16,617 HIV-positive patients showed that 7% were lost to follow-up after initiation of treatment after 6 months. In another study, of 13,726 participants whose phone number was listed, 80% were not reachable on the known phone number [6]. In a study in Cote d’Ivoire of patients on antiretroviral therapy (ART), an attempt was made to trace approximately 7000 patients through telephone calls who were lost to follow-up. Of these, only 40% of the patients could be contacted [7].

These studies raise concern about the effectiveness of telephonic follow-up as the primary method for contact and retention. There are few studies investigating the effectiveness of the use of phone numbers for follow-up in research. Insight into the frequency of nonreachability of participants and the relation with follow-up time and participant characteristics will help to develop more targeted strategies and novel ways to facilitate maintaining contact with participants in long-term follow-up studies in the future. Hence, in this study we aimed to get insight into the use of phone numbers to retain contact with participants in a study setting and evaluated determinants of reachability using a reachability questionnaire.

## Methods

### Study Population

**Figure 1:**
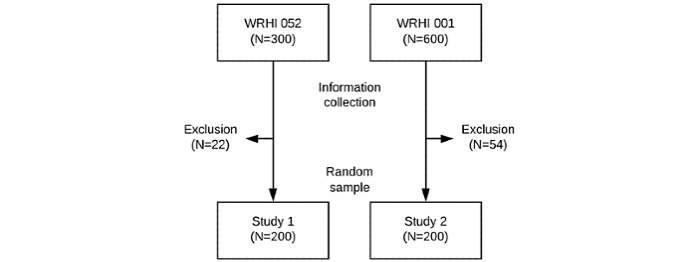
Flowchart of sample selection.

A cross-sectional study was conducted using the databases of two completed randomized controlled trials with participants diagnosed with HIV receiving ART, study 1: WRHI 052 [8] [NCT02671383] and study 2: WRHI 001 [9] [NCT02670772] (only patients recruited at the South African site were eligible for this study). The trial methods are published elsewhere [8,9]. The participants’ contact details were updated at every study visit for both trials. The latest available contact information files were used in this study. All participants in both studies consented for the use of their information and the option to be contacted for further research. Participants were excluded from this study when there were no phone numbers listed, the participant withdrew from one of the studies, or the participant was known to be deceased. Exclusion for no phone numbers listed and known deceased status resulted in 22 exclusions in study 1 and 54 exclusions in study 2. Sampling was done using the contact sheets of both studies including all participants that came for a final follow-up visit. In April 2019, a random sample of 200 participants per study was obtained (Figure 1).

The study was approved by the Human Research Ethics Committee of the University of Witwatersrand (reference number M1811107). Participation was voluntary, and informed consent was obtained verbally after explaining the study using an information sheet and answering any questions. This verbal consent was recorded using Skype for Business recording manager and securely stored digitally.

### Study Design

During the period April to June 2019, participants were contacted by phone by a single researcher. When someone answered the phone, the participant was identified by name and date of birth. Data were collected and managed using Research Electronic Data Capture (REDCap) [10]. When not reached on the first attempt, each participant was called on each available phone number to a maximum of 3 times. A participant with 3 listed phone numbers who did not answer would, therefore, be called 9 times in total. The first call attempt was made in the afternoon between 13:00 and 16:00, the second call in the morning between 9:00 and 12:00. Both calls were made from Monday to Friday. The third call was made on a Saturday between 9:00 and 16:00. No repeat calls were made on the same day, Sundays, and public holidays. If a participant did not answer after the third round, a voicemail would be left on all numbers, if this option was available, with the request to call back. Furthermore, if an email address was available a message would be sent after the third attempt on every number with a request to return the call or send an email indicating what number should be used and what time would be best for a phone call. A person could either be reached or not reached; the criteria for labeling reached or not reached can be found in Textbox 1.

**Criteria for labeling reached or not reached.**

Reached:

- Participant answered the phone, responded to voicemail, or responded to email
- Someone else (friend and/or family) answered the phone and would be able to get a message to the participant

Not reached:

- No response after three calls, voicemail, and email
- Someone else (friend and/or family) responded but would not be able to get a message to the participant

If someone else (friend and/or family) answered, this person was specifically asked if it would be possible to send the participant a message. If the phone was answered and the participant was identified, the reason for the call was explained and informed consent was obtained verbally through the phone. If the participant was fluent in English, a study-specific questionnaire was administered. In case a participant picked up the phone but was unable to complete the questionnaire due to time constraints, an additional 3 calls were made at a time that suited the participant to attempt questionnaire completion. If the participant did not answer the phone anymore, the questionnaire could not be completed but the participant was still labeled as reached.

### Questionnaire

The aim of the questionnaire (Multimedia Appendix 1) was to edit or complement the available personal information and gain more information about the reachability of the participant. The questionnaire consisted of 8 questions with subquestions to clarify a given answer. The questionnaire took around 5 to 10 minutes to complete. The questionnaire was piloted prior to data collection. The following information was checked and amended if necessary: city of residence, country of origin, and tribe or race of the participant. Assessment of reachability was completed by evaluating if all listed phone numbers were still correct and active. Phone numbers were determined to be correct when a participant or someone knowing the participant answered. Phone numbers were determined to be incorrect when the phone number was out of service or the person that answered the phone did not know the participant. The phone number status unknown was given if phone numbers were active and reachable although the phone was not answered; these phone numbers could not be identified as correct or incorrect. The following information was obtained: how many phone numbers does the participant currently have in use and why, how long has the participant been using the current phone number, has the phone number been changed and the reasons for changing phone numbers, use of email and reachability by email, and use of WhatsApp and reachability by WhatsApp. Last, the participant was asked what the best method to contact the participant would be.

### Statistical Analysis

Statistical analysis was done using SPSS Statistics version 25.0 (IBM Corporation). A *P*<.05 was considered statistically significant. Baseline characteristics were presented as means with standard deviation or as medians with interquartile ranges (IQR) in case of nonnormally distributed data. To test for differences between study 1 and study 2, an independent *t* test was used for continuous and dichotomous variables and a chi-square test was used for categorical variables. Logistic regression was used to test the effect of variables on reachability, and results were presented using odds ratios (OR) with 95% confidence intervals and *P* values. Confounders considered for reachability were age, gender, country of origin, and number of available phone numbers. Factors with a *P*<.20 in univariable analysis were included in multivariable analysis.

## Results

In total, 400 participants were called and included in this analysis. In study 1, the mean time since last visit was 0.89 (SD 0.64) years, and for study 2 this was 4.58 (SD 0.41) years (*P*<.001). The average age was significantly different between groups as participants in study 1 were on average 3.95 years older (*P*<.001). Gender and country of origin were evenly distributed between the studies. City of residence (*P*=.007) and tribe or race (*P*=.002) were both significantly different between groups with more participants residing in Johannesburg and more participants from the Zulu race in study 1 and more participants from the Ndebele race in study 2. Participants in study 1 had fewer phone numbers listed per participant. The average number of phone numbers in study 1 was 1.88 (SD 0.67) versus 2.70 (SD 0.53) in study 2 (*P*<.001). Baseline characteristics are shown in Table 1.

In total, 915 phone numbers were called for the 400 participants, and 60.3% (241/400) of participants answered the phone during the 3 rounds of calling. This outcome was significantly different between studies with a response rate of 70.5% (141/200) for study 1 and 50.0% (100/200) for study 2 (*P*<.001). Of the 241 participants who answered, after division in calling rounds, most of the participants were reached in round 1 (176/241), with decreasing numbers for rounds 2 (42/241) and 3 (23/241) with a significant difference between study 1 and study 2 (*P*<.001; Table 2).

After 3 rounds of calls, 71 voicemails were left using every phone number with voicemail available of the remaining 159 participants. Moreover, an email was sent to 15 participants that had not answered and had an email address available. Of the 71 voicemails, 2 participants returned the call, but no response was received by email.

Time since last visit was significantly associated (*P*<.001) with a decreased chance of answering the phone in univariable analysis with an OR 0.794 (95% 0.713-0.885) for each additional year (Table 3).

**Figure 2:**
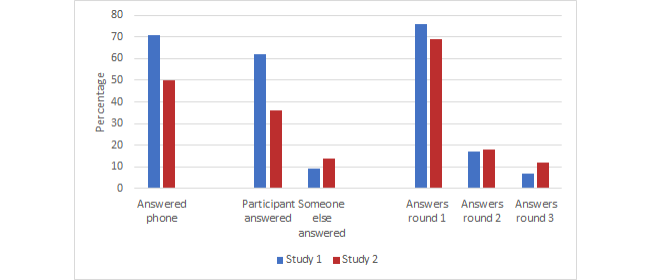
Reachability comparison between study 1 and study 2.

When the answered calls were disaggregated by participant answering the phone, someone else (friend and/or family) answering, or no answer, in study 1, 61.5% (123/200) were answered by the participant while this was 36.0% (72/200) in study 2 (*P*=.003; Figure 2).

**Table 1:** Study population characteristics.

Characteristics		Participants	Study 1 (n=200)	Study 2 (n=200)	*P* value
Age in years, mean (SD)		400	43.84 (8.1)	39.89 (7.5)	<.001
Gender, female, n (%)		400	135 (67.5)	119 (59.5)	.10
City of residence, Johannesburg, n (%)		141	79 (86.8)	42 (84)	.007
**Country of origin, n (%)**		399	200	199	.19
	South Africa	234	127 (63.5)	107 (53.8)	N/A^a^
	Zimbabwe	147	65 (32.5)	82 (41.2)	N/A
	Other	18	8 (4)	10 (5)	N/A
**Tribe or race, n (%)**		137	88	49	.002
	Zulu	48	35 (39.8)	13 (26.5)	N/A
	Ndebele	37	17 (19.3)	20 (40.8)	N/A
	Others	52	36 (40.9)	16 (32.7)	N/A
Time since enrollment in years, mean (SD)		400	2.37 (0.27)	6.16 (0.37)	<.001
Time since last visit in years, mean (SD)		400	0.89 (0.64)	4.58 (0.41)	<.001
**Listed phone numbers, n (%)**		400	200	200	<.001
	1	65	58 (29.0)	7 (3.5)	N/A
	2	155	109 (54.5)	46 (23)	N/A
	3	180	33 (16.5)	147 (73.5)	N/A

^a^N/A: not applicable.

**Table 2:** Phone call results.

Outcomes		Participants		Study 1 (n=200)		Study 2 (n=200)		*P* value	
Answered phone in total, yes, n (%)		241 (60.3)		141 (70.5)		100 (50.0)		<.001	
**Answered phone in calling round, n (%)**								<.001^a^	
	Round 1		176 (73.0)		107 (75.9)		69 (69.0)		N/A^b^
	Round 2		42 (17.4)		24 (17.0)		18 (18.0)		N/A
	Round 3		23 (9.5)		10 (7.1)		13 (13.0)		N/A
**Reachability, n (%)**								.003	
	Participant		195 (48.8)		123 (61.5)		72 (36.0)		N/A
	Someone else, friend, and/or family		46 (11.5)		18 (9.0)		28 (14.0)		N/A
	Unanswered		159 (39.8)		59 (29.5)		100 (50.0)		N/A

^a^Probability calculated using a chi-square test for the difference in answers between study 1 and 2 for all rounds.

^b^N/A: not applicable.

**Table 3:** Univariable and multivariable analysis of phone answering.

Characteristics		Univariable OR^a^ (95% CI)^b^	*P* value	Multivariable OR (95% CI)^b^	*P* value
Time since last visit		0.794 (0.713 to 0.885)	<.001	0.728 (0.631 to 0.839)	.001
Age		1.026 (1.000 to 1.053)	.047	1.018 (0.990 to 1.045)	.21
Gender, male		1.257 (0.826 to 1.912)	.29	N/A^c^	N/A
**Country of origin**					
	South Africa	Ref^d^	N/A	N/A	N/A
	Non-South Africa	0.010 (–0.092 to 0.111)	.85	N/A	N/A
**Number of phone numbers**					
	1	Ref	N/A	N/A	N/A
	2	1.650 (0.915 to 2.975)	.10	2.321 (1.236 to 4.358)	.009
	3	1.173 (0.663 to 2.075)	.58	3.038 (1.484 to 6.221)	.002

^a^OR: odds ratio.

^b^Calculated using logistic regression.

^c^N/A: not applicable.

^d^Ref: reference.

Additional adjustment for age and number of phone numbers strengthened the relation between years since last visit and reachability (OR 0.728, 95% CI 0.631 to 0.839).

The number of phone numbers listed was associated with answering the phone. Two phone numbers listed increases the odds of the participant answering the phone by OR 2.321 (95% CI 1.236 to 4.358), and three phone numbers listed increased odds by OR 3.038 (95% CI 1.484 to 6.221) with *P*=.009 and *P*=.002, respectively, compared with only having one phone number listed. Age, gender, and country of origin were not significantly related to reachability.

Of the participants who answered the phone, 72.3% (141/195) allowed the questionnaire to be completed. The other participants did not want to complete the questionnaire or they were not fluent enough in English to understand the questions. Of all the listed phone numbers, 65.4% (598/915) could be identified as either correct (383/915, 41.9%) or incorrect (215/915, 23.5%). Of the questionnaires taken, 36.2% (51/141) of participants reported using more than one phone number. The median time the questionnaire participant had been using the reached phone number was 9 (IQR 5.75-11) years. Participants often changed their number, with 64.6% (93/144) of the participants reporting having ever changed their phone number with the main reason for a change in phone number being theft of the phone, reported by 53.8% (50/93) of participants. Only 36.2% (51/141) reported having an email address and being easily reachable through it, 84.4% (119/141) reported having WhatsApp and being easily reachable on it. Of the 141 participants, 128 said the best method of connecting with them would be calling directly, followed by WhatsApp with 69 participants (Table 4).

**Table 4:** Questionnaire results.

Questionnaire outcomes		Participants, n (%)
**Questionnaires taken, n (%)**		141
	Study 1 (n=200)	92 (46.0)
	Study 2 (n=200)	51 (25.5)
**Phone numbers called (1-3 numbers per participant)**		915
	Correct^a^, n (%)	383 (41.9)
	Incorrect^b^, n (%)	215 (23.5)
	Unknown^c^, n (%)	317 (34.6)
	Use of more than 1 phone number, n (%)	51 (36.2)
	Time using current phone in years, median (IQR)^d^	9 (5.75-11)
	Reasons for change—phone stolen, n (%)	50 (53.8)
	Email user and reachable, n (%)	51 (36.2)
	WhatsApp user and reachable, n (%)	119 (84.4)
**Best method to reach (>1 answer possible), n**		
	Calling	128
	WhatsApp	69
	SMS^e^	25
	Email	11

^a^Status correct was given when a participant or someone knowing the participant answered.

^b^Status incorrect was given when the phone number was out of service or the person who answered the phone did not know the participant.

^c^Status unknown was given if phone numbers were active and reachable, but the phone was not answered; these phone numbers could not be identified as correct or incorrect.

^d^IQR: interquartile range.

^e^SMS: short message service.

## Discussion

### Principal Findings

In our study of 400 participants with varying time since the last visit to the study site, we could only subsequently reach 60.3% (241/400). None of the sociodemographic factors that were investigated showed a relation to reachability. Time since last visit and the number of phone numbers listed were the only determinants of reachability. This is the first study we are aware of that assesses the use of phone calls to stay in contact with participants in an urban African setting and how to increase reachability with these phone numbers. Phone calls are widely implemented and yet sparsely investigated.

Literature on determinants of and guidelines for retention in follow-up is scarce, but there is literature on retention percentages over time in HIV/ART studies. In a cohort study in HIV-positive patients on ART in South Africa, the incidence rate of loss to follow-up increases with time with 81.8% retention after 2 years and 54.7% retention at 5 years [11]. A systematic review of 33 patient cohorts from numerous HIV studies in sub-Saharan Africa showed that the mean retention rates were 79%, 75%, and 62% at 6, 12, and 24 months after enrollment, respectively. At 2 years, the best program retained 85% and the worst 46% [12]. Our study showed a reachability rate of 71% at 2.4 years and 50% at 6.2 years since time of enrollment. The reachability in our study is above average when compared with loss to follow-up in other studies. This might have been due to the extensive attempts to reach our participants including multiple phone calls at different times. Our questionnaire reveals a high percentage of incorrect phone numbers. Although no literature was found on the effect of the number of phone numbers per participant on reachability rate, our study shows that reachability could potentially be increased by collecting more than one phone number and by updating phone numbers at every possible occasion.

The vast majority of people who could be reached indicated a willingness to be reached via WhatsApp. WhatsApp could be an effective way to increase retention in follow-up because of the simplicity, low cost, and high percentage of users [13]. Currently, there are numerous dedicated apps that have been developed and investigated to contact participants through (push) messages to their phones [14,15]. Privacy guidelines are required for further implementation of these dedicated apps or even WhatsApp in the health care and research settings.

### Limitations

Our study comes with some limitations. We could only conduct the questionnaire if people answered the phone. Therefore, we have no information on participants who were nonreachable, and this group is crucial to better understand reasons for loss to follow-up and ways to increase retention. A second group that is underrepresented in our study is the non-English speaking group. They could not participate in the questionnaire, even though this group might have different opinions on reachability and what is important to stay in follow-up. Another limitation was related to the person who answered the call. If someone else (friend and/or family) answered but was unsure about the availability of giving a message to the participant, the call would be registered as not answered. Strengths of this study are the systematic method to evaluate phone numbers to retain participants in follow-up, the relatively large sample size, and the investigation for factors that contribute to loss to follow-up.

### Conclusion

Time since the last face-to-face visit is the main determinant for loss to follow-up in research projects in an urban African setting, while participants having more than one phone number increases the likelihood of staying in touch. The high frequency of incorrect and/or unidentified phone numbers indicates that every contact session with the participant should be used to verify and amend the available phone numbers. To further enhance reachability, the potential of WhatsApp or dedicated phone apps should be explored. Although WhatsApp was recommended as a preferred method of contact, second to phone calls, more research needs to be done to investigate what communication method is preferred by participants who did not respond to phone calls.

## Appendix

Multimedia Appendix 1 Connect Reachability Questionnaire.

## Abbreviations

ART: antiretroviral therapy
IQR: interquartile range
OR: odds ratio
REDCap: Research Electronic Data Capture
