# Supplementary material for: Phone Calls to Retain Research Participants and Determinants of Reachability in an African Setting: Observational Study
Source: JMIR Form Res. 2020 Sep 30;4(9):e19138. doi: 10.2196/19138 (PMC7557447; doi:10.2196/19138)
Supplement: Multimedia Appendix 1 [file formative_v4i9e19138_app1.docx]

## Multimedia Appendix 1

Connect Reachability Questionnaire

The following questions will be asked upon establishing contact by phone:

With the following questions I would like to get insight into your personal reachability and preferred method of establishing contact:

1. Are the other listed phone numbers such as …….(phone number 1,2 or 3) still active and correct?

Phone number 1 listed: Yes or No

Phone number 1 still active and correct: Yes or No

Phone number 2 listed: Yes or No

Phone number 2 still active and correct: Yes or No

Phone number 3 listed: Yes or No

Phone number 3 still active and correct: Yes or No

1. Do you have more than one personal number and if so why?

More than one personal phone number: Yes or No

Predefined answers if answer is yes: Extra personal phone number, extra work phone number, extra phone number for divided airtime and data, extra phone number for international calls, no specific reason

Other: if not in answer options, describe answer

1. How long have you had your current phone number?

Answer: Amount of time in years ranging between 0 and 99

1. What are the reasons for changing your phone number (if participant has indicated that they have had multiple numbers over a period of time)

Ever had a change in phone number: Yes or No

Predefined answers for change in phone number: Upgrade in phone or subscription, change in cell phone company, lost or stolen phone, no reason stated

Other: if not in answer options, describe answer

1. Are you reachable by email?

Do you have email: Yes or No

Are you easily reachable on email: Yes or No

1. Are you reachable by Whatsapp?

Do you have Whatsapp: Yes or No

Are you easily reachable on Whatsapp: Yes or No

1. Are you reachable by other methods? Such as?

Reachable by other methods: Yes or No

If yes, predefined answers for other methods: SMS, physical letter delivery

Other: if answer is not a predefined answer, describe answer

1. What is, in your opinion, the best method to stay in contact with our participants?

Predefined answers for best method: Calling, Whatsapp, SMS, email, physical letter delivery

Other: if answer is not a predefined answer, describe answer
